# Editorial: Targeting non-coding RNAs in viral disease management: potential as therapeutic targets and diagnostic biomarkers

**DOI:** 10.3389/fcimb.2026.1823469

**Published:** 2026-03-25

**Authors:** Mohsan Ullah Goraya, Muhammad Aslam, Bilal Aslam, Liaqat Ali, Bilal Ahmad Mir, Shilong Chen

**Affiliations:** 1 School of Medicine, Huaqiao University, Quanzhou, Fujian, China; 2 Bolan University of Medical and Health Sciences, Quetta, Pakistan; 3 Department of Veterinary Preventive Medicine College of Veterinary Medicine, Qassim University, Buraydah, Saudi Arabia; 4 College of Medicine, Department of Medical Physiology, Texas Agricultural and Mechanical (A&M) University, Tyler, TX, United States; 5 Department of Clinical Sciences, Faculty of Medicine, Lund University, Lund, Sweden; 6 Fujian Academy of Agricultural Sciences, Fuzhou, China

**Keywords:** diagnostic biomarkers, host-pathogen interaction, non-coding RNAs, therapeutic targets, viral infection

The virus-host arms race has created a new class of molecular regulators. Previously considered minor transcriptional byproducts, non-coding RNAs (ncRNAs) are now recognized as key viral infection regulators, offering new diagnostic and therapeutic prospects. This decade has seen significant advances in understanding how ncRNAs affect host-pathogen interactions. Epstein-Barr virus and Kaposi’s sarcoma herpesvirus use host non-coding RNAs while generating their own, enabling complex immune evasion strategies that support long-term host persistence (Media et al., 2025). The persistent danger by new and re-emerging viruses to global health security highlights the need for better diagnostic and treatment options. Recent molecular biology advances have revealed the essential roles of diverse ncRNAs, including microRNAs (miRNAs), long non-coding RNAs (lncRNAs), and circular RNAs (circRNAs), in regulating complex signalling pathways that control viral replication, immune evasion, and disease progression. As our understanding of viral infections grows, ncRNAs have become promising translational tools and demonstrated their promise role as viral disease treatment targets and diagnostic biomarkers.

Both human and viral ncRNAs can modulate the expression of host genes which are critical for viral replication, latency, activation of signalling pathways, cytokine and chemokine production, RNAi processing, expression of interferons (IFNs) and interferon-stimulated genes (ISGs) (Kesheh et al., 2022). Recent five-year study on recipients of hematopoietic stem cell transplantation indicates that persistent CMV replication, as opposed to temporary EBV reactivation, markedly elevates the risk of late mortality, underscoring the necessity for prolonged PCR monitoring for up to two years post-transplant to identify and manage patients at risk (Li et al.). The manner in which Epstein-Barr virus ncRNAs physically reorganize host chromatin is one of the most compelling discoveries. Viral ncRNAs function as “molecular sculptors,” altering the architecture of the host genome to establish viral niches, as indicated by improved three-dimensional genomics. This paradigm shift demonstrates that ncRNAs actively reorganize the nucleus and gene expression (Tian et al., 2025).

Ju et al., Reviewed that influenza A virus (IAV) subverts antiviral defences by utilizing host ncRNAs, as demonstrated by mechanistic insights. CircMerTK acts as a molecular sponge for miR-125a-3p, mitigating the suppression of targets that hinder interferon-stimulated gene expression; lnc-Lsm3b stabilizes RIG-I in an inactive conformation, impeding downstream signalling (Qiu et al., 2023). lnc-MxA forms RNA-DNA triplex structures at the IFN-β promoter, thereby directly suppressing interferon transcription; meanwhile, lncNSPL obstructs RIG-I ubiquitination by sequestering the E3 ligase TRIM25. An especially notable example is lncRNA-PAAN, which augments polymerase complex assembly and promotes viral replication by directly engaging with the viral PA protein, a crucial element of the IAV RNA-dependent RNA polymerase Ju et al. lncRNA-up4, a recently identified ncRNA, enhances avian influenza virus replication by elevating proinflammatory cytokine levels (IL-6, TNF-α, IL-1β) and inhibiting antiviral responses (IFN-β, MX1, and OAS-1) (Sajjad et al., 2025).

In addition to IAV, non-coding RNAs modulate host-virus interactions in HCV, DENV, SARS-CoV-2, HBV, HIV, and KSHV. An exhaustive examination of ncRNA-mediated regulation among diverse viral pathogens, clarifies the dual functions of these molecules as both host defence mechanisms and viral offensive agents Yamin et al.. miRNA-based treatments, particularly Miravirsen, an antisense locked nucleic acid inhibitor targeting the liver-specific pro-viral miR-122, have progressed to clinical trials for HCV infection, demonstrating the growing significance of translational applications Yamin et al.. Concurrently, diagnostic applications are progressing; circulating miRNA patterns in dengue virus infection are associated with illness progression, while lncRNA NEAT1 shows promise as a severity marker for COVID-19 Yamin et al..

A variety of integrative themes are covered in this special issue. First, as risk changes over time, temporal dynamics are important, and certain ncRNAs function as late-phase negative feedback regulators. Second, there is clear convergence on key antiviral nodes: the JAK-STAT pathway, interferon production, and RIG-I signalling are all crucial regulatory sites Ju et al. Third, universal techniques are hindered by virus-specific processes; as Yamin et al., highlighted miR-122 suppresses HBV while enhancing HCV replication, highlighting the need for pathogen-specific approaches Yamin et al.. Future research priorities including single-cell resolution analyses, improved tissue-specific delivery systems, structural characterization of ncRNAs will support logical therapeutic design, mechanistic validation using CRISPR technologies, and extensive clinical validation across various populations. Current Research Topic shows how ncRNA virology has developed from simple descriptive molecules to mechanistic analysis and shows that it is ready for therapeutic application.

## References

[B1] Kesheh M. M. Mahmoudvand S. Shokri S. (2022). Long noncoding RNAs in respiratory viruses: A review 32. doi: 10.1002/rmv.2275, PMID: 34252234 PMC8420315

[B2] Media T. S. Cano-Aroca L. Tagawa T. (2025). Non-coding RNAs and immune evasion in human gamma-herpesviruses. Viruses 17. doi: 10.3390/v17071006, PMID: 40733622 PMC12298795

[B3] Qiu H. Yang B. Chen Y. Zhu Q. Wen F. Peng M. . (2023). Influenza A virus-induced circRNA circMerTK negatively regulates innate antiviral responses 11, e03637–e03622. doi: 10.1128/spectrum.03637-22, PMID: 36847523 PMC10100971

[B4] Sajjad N. Maarouf M. Wang Y. Shrestha P. Rai K. R. Chi X. . (2025). A chicken lncRNA is identified as a critical regulator that increases influenza virus replication by impairing innate antiviral responses. Veterinary Res. 56, 195. doi: 10.1186/s13567-025-01635-4, PMID: 41094489 PMC12523108

[B5] Tian S. Z. Yang Y. Ning D. Yu T. Gao T. Deng Y. . (2025). Landscape of the Epstein-Barr virus-host chromatin interactome and gene regulation. EMBO J. 44, 3872–3915. doi: 10.1038/s44318-025-00466-5, PMID: 40425856 PMC12216251

